# Involvement of the epidermal growth factor receptor in the modulation of multidrug resistance in human hepatocellular carcinoma cells in vitro

**DOI:** 10.1186/1475-2867-11-40

**Published:** 2011-11-17

**Authors:** Katrin Hoffmann, Zhi Xiao, Clemens Franz, Elvira Mohr, Susanne Serba, Markus W Büchler, Peter Schemmer

**Affiliations:** 1 Department of General and Transplantation Surgery, Ruprecht-Karls-University, Heidelberg, Germany

## Abstract

**Background:**

Hepatocellular carcinoma (HCC) is a molecular complex tumor with high intrinsic drug resistance. Recent evidence suggests an involvement of the tyrosine kinase pathway in the regulation of ATP-binding cassette protein (ABC-transport protein) mediated multidrug resistance in cancer cells. The aim of this study was to examine whether EGFR inhibition sensitizes HCCs to chemotherapy and to elucidate its mechanism.

**Results:**

Chemotherapeutic treatment induces multidrug resistance and significantly increases ABC-transport protein expression and function in a time- and dose-dependent manner in HCC cells. Furthermore, cytostatic treatment increases the mRNA expression of tyrosine kinases and induces the phosphorylation of ERK. EGF activation of the tyrosine kinase pathway up-regulated the ABC-transport protein mRNA expression and enhanced the survival of resistant HCC cells. Consistent with these effects, inhibition of the EGFR using siRNA decreased the ABC-transport protein mRNA expression and inhibited the proliferation of resistant cells. Additional treatment with Gefitinib, a clinically approved EGFR inhibitor, caused a dose-dependent reversal of resistance to conventional chemotherapy.

**Conclusion:**

The present study demonstrates that the multidrug resistance of HCC is modulated through the EGF-activated tyrosine kinase cascade. Consequentially, the restoration of chemosensitivity by EGFR inhibition may lead towards new tailored therapies in patients with highly resistant tumors.

## Background

Hepatocellular carcinoma (HCC) is a major health problem worldwide. Its incidence is increasing continuously in the Western world. In the United States and Europe the diagnosis of HCC has almost doubled during the last two decades [1]. Despite recent improvements in surveillance programs and diagnostic tools, only 30-40% of HCC patients are eligible for liver resection or transplantation, the only curative treatment options to date [2]. The tyrosine kinase inhibitor sorafenib is the current standard of care for palliative treatment; the partial response rate, however, is only about 10% [3]. Conventional systemic chemotherapy has shown only minor effectiveness with response rates far below 10% [4]. A substantial resistance against structurally and functionally unrelated cytostatic drugs develops through the destruction of vulnerable and negative chemoresistant tumor cell populations during hepatocarcinogenesis. An increased cellular extrusion of chemotherapeutics by multidrug resistance mediating ABC-transport proteins (MDR proteins) and consequently reduced cytostatic activity has been described [5]. The expression of transmembrane ABC-transport proteins in HCC has been demonstrated *in vitro *and *in vivo *[6,7]. However, the over-expression of MDR proteins is an independent prognostic factor, associated with increased vascular and lymphatic invasion, shorter disease-free survival as well as significantly reduced overall survival [8-10].

An association of the tyrosine kinase pathway with the development and regulation of multidrug resistance (MDR) has been discussed in various tumor entities [11-14]. The epidermal growth factor receptor (EGFR)-associated activation of the tyrosine kinase pathway plays a key role in the signal transduction of cell differentiation, motility and proliferation in HCC. Guan et al. have demonstrated an increased tyrosine kinase activity in resistant hepatoma cells [15]. The induction of the tyrosine kinase activity by epidermal growth factor (EGF) and consequentially increased MDR gene expression has been discussed previously in breast cancer cells [16]. However, the involvement of the EGFR in the development of MDR in HCC has not yet been elucidated.

We show for the first time that conventional cytostatics induce the EGF-activated tyrosine kinase pathway leading to the induction of ATP-binding cassette protein mediated multidrug resistance in HCC. Moreover, we present the evidence that EGFR inhibition is a potent sensitizer for chemotherapeutic treatment in cancer cells.

## Material and methods

### HCC cell line

The human HCC cell line HepG2 (Toni Lindl GmbH, Munich, Germany) was used for *in vitro *experiments, cultured in RPMI 1640/DMEM containing 10% fetal bovine serum (FBS) in 5% CO_2 _at 37°C [17]. Cell culture reagents were obtained from Life Technologies Inc. (Gaithersburg, USA), unless indicated otherwise.

### Chemotherapeutic treatment

Gemcitabine (Lilly, Indianapolis, USA) and doxorubicin (Sandoz Pharmaceuticals GmbH, Holzkirchen, Germany) were prepared according to the manufacturer's instructions in the Pharmacy of the university hospital of Heidelberg. Gefitinib (AstraZeneca, London, UK) were prepared according to the manufacturer's instruction by dissolving in DMSO. Cells were treated as follows for induction of drug resistance: untreated controls, twice weekly, gemcitabine or doxorubicin at two different concentrations (11.4 μg/ml and 114 μg/ml or 0.15 μg/ml and 1.5 μg/ml, respectively). EGF effects were assessed in the following groups: untreated controls, EGF 500 ng/ml (Biomol, Hamburg, Germany) for 24 hours, gemcitabine in the above-mentioned doses either in combination with EGF or alone. Effects of gefitinib were analysed in the following groups: untreated controls, gefitinib 10 μg/ml, gemcitabine or doxorubicin in the above-mentioned doses in combination with gefitinib or alone.

### MTT assay

Cells at 2 × 10^3 ^were seeded into 96-well plates and cultured in 100 μl medium. Cells were treated as mentioned above, and 24, 48, 72 and 96 hours after treatment, MTT [3-(4, 4- dimeththiazol-2-yl)2, 5-diphenylterazolium bromide] in PBS was added to each well, incubated for 4 hours at 37°C and dissolved in 100 μl of propanol-2. The absorbance was recorded at 570 nm on a photometer (Eppendorf, Germany). A minimum of four independent experiments were performed in each treatment group.

### RT-PCR

Total RNA was isolated from cells and transcripted to cDNA according to the manufacturers' instructions (RNeasy Mini Kit, Qiagen, Hilden, Germany; Transcriptor First Strand cDNA Synthesis Kit, Roche Diagnostics, Basel, Switzerland). Semi-quantitative RT-PCR analysis was performed using Power SYBR Green as fluorescent probe and the StepOne RT-PCR System (Applied Biosystem, Foster City, USA). The human GAPDH was used as endogenous control. Commercially available primers for GAPDH, MRP1, MRP2, MRP3, PGP and EGFR were used (Qiagen, Hilden, Germany). Primers for RAF1 (Genbank NM002880), MEK (Genbank NM002755), ERK (Genbank NM002745) and MAPK14 (Genbank NM001315) were designed using NCBI Primer-Blast software and produced by Invitrogen, Carlsbad, USA. (Table 1) In brief, after 10 minutes of denaturation at 95°C, RT-PCR was carried out for 40 cycles at 95°C for 15 s and extension at 60°C for 60 s. The fluorescent signal was measured at the end of the annealing phase of each cycle. mRNA quantification was recorded and analyzed with the 2(-Delta Delta C(T)) method using the StepOne RT-PCR System (Applied Biosystem) [18]. All samples were measured in triplicates. Two wells were used to monitor contamination in every run. The efficiency of primers was evaluated by serial dilution of cDNA and an efficiency of ≥ 85% was reached.

**Table 1:** Primer Sequences

Protein	Forward Primer	Reverse Primer	Length (bp)
RAF1	5'- AGACTGCTCACAGGGCCTTA-3'	5'- CTGCAAATGGCTTCCTTCTC-3'	79
MEK	5'- GGTGTTTATTGGGCCTCAGA-3'	5'- ACCCGGAGCATCACAAATAG-3'	78
ERK	5'- GACAAGGGCTCAGAGGACTG-3'	5'- AGGACCAGGGGTCAAGAACT-3'	71
MAPK14	5'- TTGGTCAGTGGGATGCATAA-3'	5'- GGCTTGGCATCCTGTTAATG-3'	140

### Western blot

HepG2 cells were lysed and 40 μg of total protein was separated by electrophoresis on a SDS-PAGE and transferred to PVDF membranes using an XCell IITM Blot Module (Invitrogen, Carlsbad, USA). Blotting was performed with PBS containing 0.05% Tween 20 plus 5% BSA and incubated overnight with primary antibodies (PGP, MRP1, MRP2, MRP3, ERK all Santa Cruz Biotechnology Inc., Santa Cruz, USA, EGFR and pERK Cell signalling Technology, Danvers, MA, USA, Actin Sigma Aldrich, Munich, Germany) at 4°C. The horseradish-peroxidase conjugated secondary antibody (Santa Cruz) was used for protein detection at room temperature for 2 hours, followed by chemiluminescence detection (ECL Western blot Analysis System, GE Healthcare, Munich, Germany). The results of Western blot were analyzed with the analysis software QUANTITY ONE (BIO-RAD Laboratories, Hercules, CA, USA).

### EGFR inhibition

EGFR small interfering RNA (siRNA) duplexes that target the sequences 5'-TACGAATATTAAACACTTCAA-3' and high-purity positive control siRNA oligo-nucleotides were used for siRNA experiments following the manufacturer's instructions (Qiagen, Hilden, Germany). Cells at 4 × 10^4 ^were seeded in the 6-well plates and transfection was performed using 12.5 nM siRNA and 12 μl transfection reagent according to the manufacturer's protocol (HiPerfect Transfection Reagent, Qiagen). After 48 hours, total RNA was extracted from cells and RT-PCR performed as described above.

### Rhodamine uptake assay

Rhodamine uptake assay was performed to evaluate the PGP transport function. Cells were treated as mentioned above and incubated with PBS, Verapamil or Rhodamine 123. Rhodamine uptake was measured with FACS Canto II Flow Cytometry System (Becton Dickinson, New York, USA) and analyzed by FACS Diva 6.0 software as described by Huet et al. [19].

### Statistics

A one-way Anova test was carried out to reveal significant differences in the mRNA expression. A value of p ≤ 0.05 was defined as the level of significance. All statistical analyses were performed with SigmaStat 1.0 software (Jandel Scientific, Sanrafael, CA, USA).

## Results

### Induction of multidrug resistance after chemotherapy

**Figure 1:**
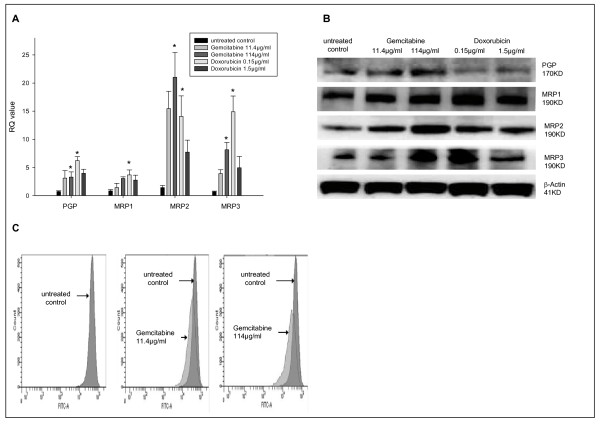
**Chemotherapy induces multidrug resistance in HCC cells**. Gemcitabine (11.4 μg/ml or 114 μg/ml) or Doxorubicin (0.15 μg/ml or 1.5 μg/ml) was added to HepG2 cells twice weekly and cultured in RPMI 1640 containing 10% FBS. A: mRNA expression of ATP-binding cassette proteins was assessed by RT-PCR. Columns average of three independent experiments, bars SD. B: protein expression of ATP-binding cassette proteins was assessed by Western blot. C: PGP transport function was assessed by Rhodamine uptake assay and analysed by FACS. *p ≤ 0.05 compared to the untreated control group.

The effect of conventional chemotherapy on MDR protein expression and function was analyzed by RT-PCR, Western blot and Rhodamine uptake assay. PGP-, MRP1-, MRP2- and MRP3-mRNA was detected by RT-PCR in untreated HepG2 Chemotherapy induced multidrug resistance in HepG2 cells. After treatment with gemcitabine, a significant dose-dependent increase of ABC-transport protein mRNA expression was detectable compared to the control group (p ≤ 0.05). After treatment of HepG2 cells with gemcitabine 114 μg/ml the MRP2-, MRP3- and PGP-mRNA levels were increased fourteen-, eleven- and four-fold, respectively, compared to the control group (p ≤ 0.05). Doxorubicin treatment lead to an enhanced mRNA detection compared to controls (p ≤ 0.05). The levels of MRP1- MRP2-, MRP3- and PGP-mRNA increased two-, nine-, twenty- and seven-fold, respectively, after treatment with doxorubicin 0.15 μg/ml (p ≤ 0.05) (Figure 1a). On a protein level, a dose-dependent increase of MRP2 and PGP expression was detected after Gemcitabine treatment by Western blot analysis. Gemcitabine at a dose of 11.4 μg/ml increased the MRP2 protein levels by 43% and Gemcitabine at a dose of 114 μg/ml by 76% compared to the control group (p ≤ 0.05). Furthermore, PGP protein levels increased by 20% after treatment with Gemcitabine 11.4 μg/ml and 139% after Gemcitabine at a dose of 114 μg/ml (p ≤ 0.05) (Figure 1b). A dose-dependent higher PGP activity was detectable in the rhodamine uptake assay after treatment with gemcitabine (Figure 1c).

### Chemotherapy-induced effects on the tyrosine kinase pathway

**Figure 2:**
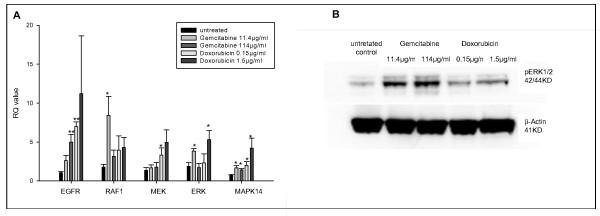
**Chemotherapy induces the tyrosine kinase cascade mRNA expression in HCC cells**. Gemcitabine (11.4 μg/ml or 114 μg/ml) or Doxorubicin (0.15 μg/ml or 1.5 μg/ml) was added to HepG2 cells twice weekly and cultured in RPMI 1640 containing 10% FBS. A: Tyrosine kinase mRNA expression was assessed by RT-PCR. Columns average of three independent experiments, bars SD. B: protein expression of tyrosine kinases was assessed by Western blot. *p ≤ 0.05; **p ≤ 0.001 compared to the untreated control group.

The effect of conventional chemotherapy on the mRNA expression levels of various tyrosine kinases was analyzed after induction of MDR. Chemotherapy induced the expression of tyrosine kinase pathway associated mRNAs in HepG2 cells. Gemcitabine increased mRNA levels of RAF1, ERK, MAPK14 and EGFR significantly (p ≤ 0.05). The expression of RAF1, ERK and EGFR increased two- to three-fold of control values in a dose-dependent manner. Doxorubicin lead to a dose-dependent two- to six-fold increase of RAF1-, ERK- and MAPK14-mRNA expression compared to controls (p ≤ 0.05) (Figure 2a). Furthermore, treatment with gemcitabine or doxorubicin lead to a dose-dependent increase of pERK protein expression by 3- and 2-folds, respectively, compared to the untreated controls (Figure 2b).

### Effects of EGF on multidrug resistance proteins

**Figure 3:**
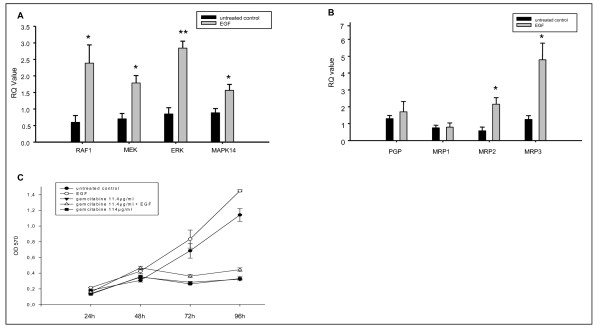
**EGF stimulated activation of the tyrosine kinase pathway increases the ABC-transport protein mRNA expression and cellular proliferation of resistant HepG2 cells**. Resistant HCC cells were cultured in RPMI 1640 and treated with EGF 500 ng/ml for 24 hours. A: Tyrosine kinase mRNA expression was assessed by RT-PCR. Columns average of three independent experiments, bars SD. B: ABC-transport protein mRNA expression was assessed by RT PCR. Columns average of three independent experiments, bars SD. C: Cellular viability was analyzed by MTT assay. A minimum of three independent experiments was performed. *p ≤ 0.05 compared to the untreated control group.

To investigate whether an activation of the tyrosine kinase pathway would change the drug resistance phenotype, the effects of EGF on drug resistant cells were evaluated with RT-PCR and MTT assay. EGF increased the mRNA expression of RAF1, MEK, ERK and MAPK14 significantly in HepG2 cells (Figure 3a). Furthermore, EGF induced a significant increase of MDR protein mRNA expression in HepG2 cells. The mRNA levels of MRP2 and MRP3 were three-fold higher in HepG2 cells after EGF treatment compared to the controls (p ≤ 0.05) (Figure 3b). Survival of resistant cells was increased after activation of the tyrosine kinase pathway in a time-dependent manner. Ninety-six hours after EGF the survival of gemcitabine-treated cells was 10-13% higher compared to cells treated with gemcitabine alone (p ≤ 0.05) (Figure 3c).

### Involvement of the EGFR in the regulation of multidrug resistance

**Figure 4:**
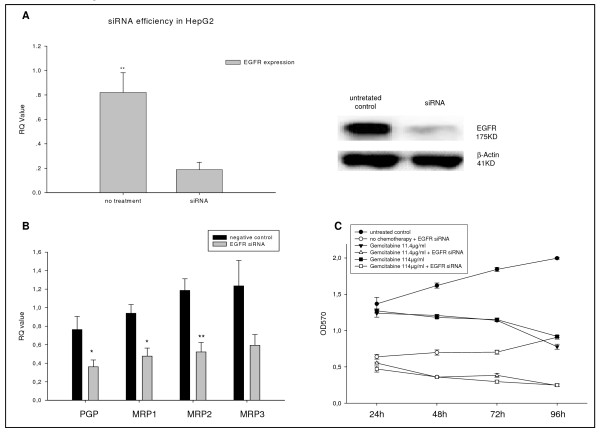
**EGFR inhibition by siRNA reduces the ABC-transport protein mRNA expression and cellular proliferation of HepG2 cells**. A: HepG2 cells were transfected with EGFR siRNA. EGFR mRNA expression was assessed by RT-PCR. Columns average of three independent experiments, bars SD. EGFR protein expression analysed by Western blot. B: ABC-transport protein mRNA expression was determined by RT PCR in HepG2 cells treated with EGFR siRNA. Columns average of three independent experiments, bars SD. *p ≤ 0.05; **p ≤ 0.001 compared to the untreated control group. C: HepG2 cells were treated with EGFR siRNA, Gemcitabine 11, 4 μg/ml, Gemcitabine 11, 4 μg/ml plus EGFR siRNA, Gemcitabine 114 μg/ml and Gemcitabine 114 μg/ml plus EGFR siRNA. Cellular viability was analyzed by MTT assay. A minimum of three independent experiments was performed.

To evaluate whether the EGF receptor is involved in the regulation of MDR, siRNA was used to inhibit its expression. An inhibition efficiency of 85.5% was obtained (Figure 4a). After EGFR inhibition, the mRNA levels of multidrug resistance proteins were significantly decreased in resistant cells. Inhibition of EGFR in gemcitabine-treated cells decreased PGP-, MRP1- and MRP2-mRNA levels by 53%, 49% and 56%, respectively, compared to cells with EGFR intact (p ≤ 0.05) (Figure 4b). Furthermore, the mRNA levels of both RAF1 and MAPK14 were significantly lower after EGFR inhibition and chemotherapy compared to resistant cells with EGFR intact (p ≤ 0.05) (data not shown). Altogether, the inhibition of the EGFR lowered the survival rate to 50% compared to cells with EGFR intact (p ≤ 0.05). EGFR inhibition in chemotherapy-treated resistant cells decreased survival to 75% compared to resistant cells with EGFR intact (p ≤ 0.001) (Figure 4c).

### Effects of the specific EGFR inhibitor gefitinib on multidrug resistance

**Figure 5:**
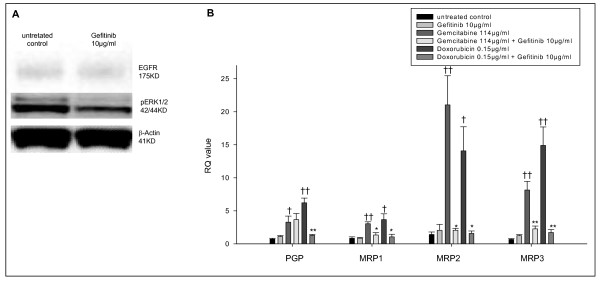
**Gefitinib treatment restores the chemosensitivity in HepG2 cells**. A: HepG2 cells were treated with Gefitinib 10 μg/ml and EGFR as well as pERK protein expression was assessed by Western blot. B: HepG2 cells were treated with Gefitinib 10 μg/ml, Gemcitabine 114 μg/ml, Gemcitabine 114 μg/ml plus Gefitinib 10 μg/ml, Doxorubicin 0.15 μg/ml and Doxorubicin 0.15 μg/ml plus Gefitinib 10 μg/ml. ABC-transport protein mRNA expression was determined by RT PCR. Columns average of three independent experiments, bars SD. ^†^p ≤ 0.05; ^††^p ≤ 0.001 compared to the untreated control group; *p ≤ 0.05; **p ≤ 0.001 compared to monotherapy.

To evaluate whether the addition of a clinically approved EGFR inhibitor to conventional chemotherapy might restore chemosensitivity, the effects of both, gemcitabine and doxorubicin plus gefitinib were analyzed. Gefitinib monotherapy did not change the EGFR protein expression but reduced the pERK expression (Figure 5a). While the mRNA expression of PGP, MRP1, MRP2 or MRP3 did not change after gefitinib monotherapy, a simultaneous treatment of gefitinib and conventional chemotherapy significantly restored the chemosensitivity in HepG2 cells. The combination of gemcitabine plus gefitinib reduced the MRP1-, MRP2- and MRP3-mRNA levels two-, ten- and four-fold, respectively, compared to monotherapy with gemcitabine (p ≤ 0.05). Doxorubicin plus gefitinib decreased the PGP-, MRP1-, MRP2 and MRP3-mRNA levels five-, three- nine- and nine-fold compared to monotherapy with doxorubicin (p ≤ 0.05) (Figure 5b).

## Discussion

Hepatocellular carcinoma is a molecular complex tumor with high intrinsic drug resistance [20]. New approaches to overcome this resistance and offer patients tailored treatment strategies are urgently required [21]. In this study we investigated the ability of tyrosine kinase inhibition to restore chemosensitivity in HCC. We demonstrate for the first time that EGFR inhibition sensitizes HCC cells to conventional chemotherapy. Furthermore, we provide evidence that EGFR-activated signal transduction via the tyrosine kinase pathway is involved in the development of MDR in HCC.

Indeed, data presented in this study clearly show that standard chemotherapy dramatically induces MDR in both of the investigated HCC cells. Both gemcitabine and doxorubicin treatment significantly increased the ABC-transport protein expression and mRNA levels in a time- and dose dependent manner. Additionally, cytostatic treatment enhanced the PGP activity. Thus the survival of drug resistant cells was significantly prolonged compared to chemo-sensitive cells. This is in line with previous reports, demonstrating an up-regulation of ABC-transport proteins in HepG2 cells as well as in patients with HCC after chemotherapy [15,22]. The over-expression of drug-resistance proteins is an independent prognostic factor for the impaired survival of HCC patients and conventional chemotherapy has shown only minor effectiveness, with low response rates of 5-10% [4,8-10].

There is upcoming evidence of a potential link between the tyrosine kinase pathway and ABC-transport proteins. Previously, cisplatin-induced ERK activation was described in human cervical carcinoma cells [23]. However, several factors may be responsible for the modulation of the drug-resistance phenotype and the regulatory mechanisms involved have yet not been identified [5]. Up to now an increased phosphorylation of ABC transporters by activation of the EGFR-RAS-MAPK cascade or modulation of the MDR transporter ATPase activity due to tyrosine kinase inhibition have been discussed [12,24]. In the present study, we found that chemotherapeutic treatment influenced the gene expression of tyrosine kinases. The mRNA levels of RAF1, ERK, MAPK14 and the EGFR increased in a dose-dependent manner after treatment with gemcitabine or doxorubicin. Furthermore, chemotherapy enhanced the activity of ERK and increased the protein expression of its phosphorylated form in a dose-dependent manner which is in line with a previous report of Wang et al. [23].

To test the hypothesis of an interaction between the tyrosine kinase pathway and MDR we activated the EGFR-RAS-MAPK cascade by EGF. A simultaneous increase of MDR protein mRNA expression was found after EGF treatment in both of he investigated HCC cell line, with dramatically increased gene expression levels of PGP, MRP2 and MRP3 mRNA. In line with this, PGP efflux activity was enhanced and the cellular survival significantly increased in a time-dependent manner. Simultaneously, the gene expression of EGF-activated tyrosine kinases increased. These observations are consistent with previous reports on a potential influence of EGF on PGP and MRP1 expression [16,25,26]. An EGF-stimulated activation of the EGFR and increased PGP protein expression were described in colorectal cancer cells by Katayama et al. [25]. Furthermore, enhanced MRP1 gene expression and a high MRP1 promoter activity have been detected in the presence of EGF in MCF-7 breast cancer cells [16].

Since our data indicate an involvement of the EGF-mediated downstream activation of tyrosine kinases in the regulation of ABC-transport proteins, we inhibited the EGFR using siRNA. Consequentially, an increased cytotoxicity of conventional chemotherapy and reduced survival of resistant cells was detectable. The ABC-transport protein gene expression was found to be significantly lower after EGFR inhibition in these cells. This supports the report of Garcia et al., who described a decreased MRP1 expression after inhibition of the EGFR in breast cancer cells for the first time [16]. In addition, since the EGFR is over-expressed in several highly resistant tumor entities and restoration of chemosensitivity might have a significant therapeutic impact, we evaluated the effects of gefitinib as a commercially available EGFR inhibitor on the drug-resistance phenotype [27-29]. Gefitinib is FDA approved for the treatment of advanced non-small cell lung cancer and attaches to the ATP-binding site of the EGFR. This study clearly demonstrates considerable chemosensitizing effects of combinative treatment with gefitinib in resistant hepatocellular carcinoma cells. The ABC-transport protein gene expression levels dropped by up to ten-fold after addition of gefitinib to gemcitabine or doxorubicin treatment. In line with this, increased growth inhibitory activity was detected and the cellular efflux function of PGP was reduced. Recently, a dose-dependent reversal of drug resistance in breast and lung carcinoma cell lines after simultaneous treatment with clinically relevant doses of gefitinib has been shown [30]. Furthermore, Gaikwad et al. detected decreased PGP-mRNA levels after combinative treatment with gefitinib and cisplatin in endometrial cancer cells [31]. Nevertheless, synergistic effects of gefitinib and chemotherapeutic agents have yet not been observed in clinical trials [32,33].

## Conclusions

In conclusion, the EGF-activated tyrosine kinase pathway seems to be involved in the regulation of MDR in HCC. The tyrosine kinase mRNA expression and phosphorylation is up-regulated in resistant HCC cells. Furthermore, the gene expression and function of ABC-transport proteins can be induced by EGFR activation. In contrast, the inhibition of the EGFR restores the chemosensitivity of drug-resistant HCC cells. In terms of a clinical perspective, the combination of EGFR inhibitor and selected conventional chemotherapeutic agents may be a novel strategy to improve the treatment efficacy of tailored therapies in a variety of patients with highly resistant tumors.

## Competing interests

The authors declare that they have no competing interests.

## Authors' contributions

KH and XZ performed the experiments. KH designed the study, collected and analyzed the data and wrote the manuscript. SS contributed to the experiments, EM gave the technical support, MWB and PS wrote and revised the manuscript. All authors read and approved the final manuscript.
